# Impact of enhanced recovery after surgery protocols on post-operative outcomes in elective abdominal surgeries: A prospective study

**DOI:** 10.6026/973206300223461

**Published:** 2026-06-30

**Authors:** Irfan Ahmad Siddiqui, Sushil Chand Verma, Ajita Tiwari

**Affiliations:** 1 Department of Anaesthesia, Government Medical College, Satna, Madhya Pradesh, India; 2 Department of Anaesthesia, Birsa Munda Government Medical College, Shahdol Madhya Pradesh, India

**Keywords:** Enhanced recovery after surgery (ERAS), post-operative recovery, abdominal surgery, complications, hospital stay

## Abstract

Enhanced Recovery after Surgery (ERAS) is an evidence-based perioperative approach designed to minimize surgical stress and enhance post- operative recovery. Therefore, it is of interest to evaluate the impact of ERAS protocols on post-operative outcomes in patients undergoing elective abdominal surgeries. This prospective comparative study included 100 patients who were divided into ERAS and conventional care groups, with assessment of recovery parameters, pain scores, complications and hospital stay. Patients managed with ERAS protocols demonstrated significantly reduced hospital stay, faster return of bowel function, lower post-operative pain scores and fewer complications compared to conventional care. Thus, data shows that ERAS protocols are effective in improving post-operative outcomes and should be considered as a standard perioperative care strategy in abdominal surgeries.

## Background:

Elective abdominal surgeries are among the most commonly performed surgical procedures worldwide and include a broad spectrum of operations involving the gastrointestinal, hepatobiliary, colorectal and gynecological systems [1]. Despite advances in surgical techniques, anesthesia and perioperative care, post-operative complications remain a significant concern and contribute substantially to patient morbidity, prolonged hospitalization, delayed recovery, increased healthcare costs and reduced quality of life [2]. Traditional perioperative management practices, characterized by prolonged pre-operative fasting, extensive bowel preparation, liberal fluid administration, delayed oral feeding and extended bed rest, have been associated with increased surgical stress responses and slower post-operative recovery [3]. Consequently, there has been a growing emphasis on developing evidence-based strategies that optimize perioperative care and improve surgical outcomes [4]. Enhanced Recovery After Surgery (ERAS) is a multidisciplinary, multimodal perioperative care pathway designed to minimize the physiological and psychological stress associated with surgery while promoting faster recovery [5]. Initially introduced by Kehlet in the late 1990s, ERAS protocols integrate various evidence-based interventions across the pre-operative, intraoperative and post-operative phases of patient management [6]. These interventions include comprehensive patient education, shortened fasting periods, pre-operative carbohydrate loading, optimized anesthetic techniques, goal- directed fluid therapy, minimally invasive surgical approaches, multimodal analgesia with reduced opioid dependence, early mobilization and early resumption of oral nutrition. By addressing multiple factors that contribute to post-operative morbidity, ERAS aims to enhance patient recovery without compromising safety [7]. The physiological rationale behind ERAS is based on reducing the metabolic and inflammatory responses induced by surgical trauma [8]. Surgical stress triggers neuroendocrine and inflammatory pathways that can result in insulin resistance, impaired immune function, catabolism and delayed restoration of normal physiological functions [9]. ERAS protocols seek to attenuate these responses through coordinated perioperative interventions, thereby preserving organ function and promoting a more rapid return to baseline health status [10]. Numerous studies have demonstrated that patients managed under ERAS pathways experience improved clinical outcomes compared with that receiving conventional perioperative care [11]. Over the past two decades, ERAS programs have gained widespread acceptance across various surgical specialties, particularly in colorectal and other abdominal surgeries [12]. Several randomized controlled trials, systematic reviews and meta- analyses have reported reductions in post-operative complications, length of hospital stay, post-operative pain and healthcare expenditures among patients treated using ERAS protocols [13]. Furthermore, these benefits have been achieved without increases in readmission rates, reoperation rates, or mortality, highlighting the safety and effectiveness of this approach [14]. As healthcare systems increasingly focus on value-based care and resource optimization, ERAS has emerged as a cornerstone of modern perioperative management [15]. Therefore, it is of interest to evaluate the influence of enhanced recovery after surgery protocols on post-operative outcomes in elective abdominal surgeries, with particular emphasis on post-operative complications, recovery parameters, length of hospital stay, patient satisfaction and overall clinical effectiveness.

## Methods:

This prospective comparative study was conducted over a period of one year at a tertiary care center. A total of 100 patients undergoing elective abdominal surgeries were enrolled and divided into two groups: ERAS group (n = 50) and conventional care group (n = 50). Patients were allocated using a non-randomized sequential allocation method to maintain balanced group distribution.

## Inclusion criteria:

[1] Age between 18 and 70 years

[2] Patients scheduled for elective abdominal surgery

[3] ASA physical status I-III

[4] Willingness to participate in the study

## Exclusion criteria:

[1] Emergency surgical cases

[2] Patients with severe systemic illness (ASA IV or above)

[3] Pregnant patients

[4] Patients with prior major abdominal surgeries influencing recovery

## ERAS protocol implementation:

Patients in the ERAS group received standardized perioperative care including pre-operative counseling, reduced fasting duration and carbohydrate loading where appropriate. Multimodal analgesia with opioid-sparing techniques was used, along with early post-operative mobilization and initiation of oral feeding within 24 hours.

## Outcome measures:

The primary outcomes included length of hospital stay, time to return of bowel function, post-operative pain using the Visual Analog Scale (VAS), post-operative complications and patient satisfaction.

## Statistical analysis:

Data were analyzed using SPSS version 25. Continuous variables were expressed as mean ± standard deviation, while categorical variables were presented as frequencies and percentages. Independent t-test and chi-square test were applied as appropriate. A p-value of <0.05 was considered statistically significant.

## Results:

The present study included a total of 100 patients who were equally distributed between the ERAS group and the conventional care group. Both groups were analyzed and compared across multiple perioperative parameters to evaluate the effectiveness of ERAS protocols (Table 1). The two groups were comparable in terms of baseline demographic characteristics, indicating appropriate group allocation. Patients in the ERAS group showed significantly faster recovery across all measured parameters (Table 2). ERAS protocol was associated with significantly lower post-operative pain scores (Table 3). A notable reduction in overall complications was observed in the ERAS group. Patient satisfaction was also found to be higher in the ERAS group compared to the conventional care group (Table 4). ERAS group showed a significantly shorter hospital stay compared to the conventional group, indicating faster post-operative recovery (Figure 1 - see PDF). ERAS group demonstrated a faster reduction in post-operative pain scores compared to the conventional group, reflecting better pain control (Figure 2 - see PDF).

## Discussion:

Enhanced Recovery After Surgery (ERAS) protocols have transformed perioperative care by integrating evidence-based interventions aimed at reducing surgical stress, accelerating recovery and improving post-operative outcomes. These multimodal pathways encompass pre-operative counseling, optimized nutrition, minimal fasting, standardized anesthesia, early mobilization and early enteral feeding. The growing body of literature demonstrates that ERAS protocols significantly improve recovery following elective abdominal surgeries while maintaining patient safety. Ljungqvist *et al.* (2017) [16] emphasized that ERAS pathways reduce the physiological stress response associated with surgery and facilitate the preservation of post-operative organ function. Their review highlighted that successful implementation of ERAS principles leads to reduced post-operative morbidity; shorter hospital stays and improved patient satisfaction. The authors further noted that compliance with ERAS elements is directly associated with better clinical outcomes. Similarly, Gustafsson *et al.* (2019) [17] reported that adherence to ERAS recommendations in elective colorectal surgery resulted in significant reductions in post-operative complications and duration of hospitalization. Their guidelines stressed the importance of multimodal perioperative care, including carbohydrate loading, opioid-sparing analgesia, goal-directed fluid therapy and early mobilization, all of which contribute to enhanced post-operative recovery. Lee *et al.* (2020) [18] conducted a meta-analysis evaluating ERAS protocols in gastrointestinal surgery and demonstrated significantly shorter hospital stays and faster return of bowel function among patients managed under ERAS pathways. The study also reported reduced post-operative pain and fewer overall complications compared with conventional perioperative care, supporting the widespread adoption of ERAS principles in abdominal surgical procedures. Parthsarthi *et al.* (2025) [19] analyzed randomized controlled trials involving laparoscopic digestive system surgeries and found that ERAS programs were associated with earlier ambulation, quicker gastrointestinal recovery and lower post-operative complication rates. We concluded that ERAS protocols are both safe and effective, offering substantial benefits without increasing readmission rates or mortality. More recently, Sauro *et al.* (2024) [20] reported that ERAS implementation across multiple surgical specialties consistently reduced post-operative complications, length of hospital stay and healthcare costs. Their findings underscored the broad applicability of ERAS pathways and highlighted the importance of institutional commitment and multidisciplinary collaboration to achieve optimal outcomes. Collectively, the available evidence indicates that ERAS protocols substantially improve post-operative outcomes in elective abdominal surgeries. Consistent benefits include reduced post-operative pain, earlier return of bowel function, shorter hospital stay, decreased complication rates and improved patient satisfaction. Although variations in protocol adherence and institutional resources may influence outcomes, the overall literature strongly supports ERAS as the current standard of perioperative care for elective abdominal procedures. Future research should focus on enhancing protocol compliance, evaluating long-term outcomes and adapting ERAS principles to diverse healthcare settings to maximize patient benefits [21, 22].

## Conclusion:

The implementation of ERAS protocols in elective abdominal surgeries significantly improves post-operative outcomes by reducing hospital stay, accelerating recovery, minimizing complications and enhancing patient comfort. Adoption of ERAS should be encouraged as a standard perioperative care approach to optimize surgical outcomes and improve healthcare efficiency.

## Figures and Tables

**Table 1:** Baseline Characteristics of study population

**Variable**	**ERAS Group**	**Conventional Group**	**p-value**
Mean Age (years)	45.2 ±10.5	46.8 ± 11.2	0.48
Male (%)	60%	58%	0.82
Female (%)	40%	42%	0.82

**Table 2:** Comparison of post-operative recovery parameters

**Parameter**	**ERAS Group**	**Conventional Group**	**p-value**
Hospital Stay (days)	5.2± 1.2	7.1± 2.0	<0.001
Time to Bowel Recovery (days)	1.3± 0.5	2.4± 0.8	<0.001
Time to Mobilization (days)	1.2± 0.4	2.8± 0.9	<0.001

**Table 3:** Post-operative pain assessment (VAS Score)

**Time Point**	**ERAS Group**	**Conventional Group**	**p-value**
Post-operative Day 1	4.2 ± 1.1	6.5 ± 1.3	<0.001
Post-operative Day 3	2.8 ± 0.9	4.9 ± 1.0	<0.001

**Table 4:** Post-operative complications

**Complication**	**ERAS (%)**	**Conventional (%)**	**p-value**
Surgical Site Infection	6%	14%	0.18
Post-operative Ileus	4%	16%	0.04
Overall Complications	10%	30%	0.01
